# Equitable and Feasible Distribution of SARS-CoV-2 Vaccines for All in Africa

**DOI:** 10.4269/ajtmh.21-0264

**Published:** 2021-06-28

**Authors:** Abu Bakarr Rogers, Mohamed Bailor Barrie, Mosoka P. Fallah, J. Daniel Kelly

**Affiliations:** 1 School of Medicine, Stanford Medical School, Stanford, California;; 2 Institute for Global Health Sciences, University of California, San Francisco, San Francisco, California;; 3 Partners In Health–Sierra Leone, Freetown, Sierra Leone;; 4 U.S. Partnership for Research on Ebola Virus in Liberia (PREVAIL), Monrovia, Liberia;; 5 A.M. Dogliotti College of Medicine, University of Liberia, Monrovia, Liberia;; 6 Department of Epidemiology and Biostatistics, University of California, San Francisco, San Francisco, California;; 7 San Francisco VA Medical Center, San Francisco, California;; 8 F.I. Proctor Foundation, University of California, San Francisco (UCSF), San Francisco, California

## Abstract

As the fight against the coronavirus disease 2019 (COVID-19) pandemic continues, the necessity for wide-scale, global vaccine rollout to reduce the spread of severe acute respiratory syndrome coronavirus 2 (SARS-CoV-2) and slow its mutation rate remains unassailable. The COVID-19 Vaccines Global Access (COVAX) initiative’s campaign involves a proportional framework to finance and distribute SARS-CoV-2 vaccines in low- and middle-income countries. However, the COVAX framework has critical limitations, including limited funding and the failure to account for the special epidemic risks and needs of its participating nations, as recommended by the World Health Organization’s Strategic Advisory Group of Experts on Immunization framework. These drawbacks disproportionately impact Africa, where many nations rely on COVAX as their main source of vaccines. The current plan to vaccinate only up to 20% of participating nations’ populations is short-sighted from both epidemiologic and moral perspectives. COVAX must commit to vaccinating all of Africa and its initiative must be modified to account for the health and economic infrastructures in these countries. Lessons learned from successful vaccination campaigns, including the West African Ebola outbreak, have shown that vaccinating all of Africa is possible and feasible, and that infrastructure and human resources can support mass vaccination. To halt this global pandemic, global responsibility must be accepted to finance and equitably distribute SARS-CoV-2 vaccines to African nations. We urge COVAX to act swiftly to prevent Africa from becoming the new face of a persisting pandemic.

To end the coronavirus disease 2019 (COVID-19) pandemic, we must reach worldwide community immunity. As of May 17, 2021, 1.47 billion vaccines have been administered worldwide, with 1.8 doses administered per 100 people in Africa compared with 54 per 100 people in North America.^1^ Only 0.3% of the 1.47 billion doses have been administered in low-income countries.^1^ The ability of high-income countries to administer vaccines at a much faster pace than many African nations highlights the inequities in the commitment to global vaccination. If this continues, then the persisting frontier of the COVID-19 war will be Africa, where the virus will continue to mutate and decrease vaccine effectiveness until we need another booster; for example, the 501Y.V2 variant in South Africa is highly transmissible and has spread to other nations.^2^ The international COVID-19 Vaccines Global Access (COVAX) initiative was formed in 2020 to address the pressing need for severe acute respiratory syndrome coronavirus 2 (SARS-CoV-2) vaccines in low- and middle-income countries (LMICs).^3^ With more than 190 participating countries, COVAX plans to finance and distribute two billion vaccine doses, with the goal of vaccinating up to 20% of participating countries’ populations.^3^ This is a welcome first step, but it is not an equitable solution, especially for African nations that must rely on COVAX as their main source of vaccines. It is necessary and feasible to vaccinate all of Africa to end this pandemic. COVAX should immediately commit to providing 100% coverage to low-income African nations. Wealthy nations should help fund this effort not only because it is an ethical imperative but also because it is a matter of self-interest; as community immunity decreases, the possibility of generating new and dangerous variants increases.^4^ However, for COVAX to approach community immunity or 100% coverage, major changes must be made to its current framework.

Since its inception, COVAX has struggled to achieve its primary goal of providing emergency vaccine relief in a timely manner. The COVAX initiative anticipates a two-phase mechanism for its vaccine allocation to ensure “fair” distribution. As of May 17, 2021, COVAX has shipped only 67 million vaccines to 124 countries; however, 1.47 billion vaccines have been administered worldwide.^5^ Less than 4% of the reported confirmed vaccine purchases have been designated to countries in the African union, with countries like Nigeria reporting that less than 1% of its population is vaccinated.^6^ The problem is multifaceted and calls for multimodal, multinational responses. The short-sighted goal to vaccinate up to 20% of the African population would never be an acceptable benchmark in high-income countries. Although it is true that more doses will be allocated eventually based on the needs of the nations,^7^ COVAX has been stymied in providing the number of vaccines needed to reach 20% because of insufficient funding, vaccine hoarding in high-income countries, and limited capacity to produce vaccines globally as a result of the assertion of intellectual property rights. The tepid advocacy for the waiver or transfer of these rights has made it difficult for some LMICs to produce their own vaccines.^8^ Several solutions have been proposed, including utilizing sustainable development goals to fund vaccines^9^ and the transfer of intellectual property to increase vaccine production in LMICs.^8^ However, COVAX has failed to incorporate long-term, sustainable solutions in its framework. It should commit to equipping participating nations with the skills and resources needed to produce their vaccines and to investing in the vaccine storage and distribution infrastructure. Because of the immediate need for vaccine boosters, there needs to be a sustained distribution plan.

Concerns have been raised that the infrastructure in some African countries cannot support vaccine storage and distribution, and that there may be vaccine hesitancy. These anecdotal beliefs dismiss the lessons learned from successful community-based vaccination campaigns implemented in the past and other public health initiatives. In addition to the Ebola vaccination campaigns, long-standing programs include the pan-African maternal and child health campaigns, several vaccination campaigns through the Expanded Program on Immunization, and the control and eradication of diseases like smallpox.^10^ In Sierra Leone, the Ebola vaccine required a −80° cold chain, like the Pfizer COVID-19 vaccine (New York, NY). The international community successfully delivered vaccines to rural areas around the affected villages using portable storage coolers and achieved the goals and primary endpoints of the campaign. Sierra Leone’s community-based intervention built trust and engaged the community in further trials and vaccine distribution.^11^ In 2020, the Democratic Republic of Congo experienced the world’s second deadliest Ebola outbreak. The Ebola vaccine was successfully deployed in the midst of the outbreak and more than 300,000 people were vaccinated; this was crucial to combatting the epidemic.^12^ These country case studies exemplify our contention that it is absolutely feasible to vaccinate all of Africa and addresses concerns about implementation and vaccine hesitancy. Although we urge COVAX to invest in an ultra-cold chain infrastructure, the infrastructure should not be thought of as bricks and mortar; it could also be mobile, using cooling vehicles and portable storage coolers. The current infrastructure in Africa can support mass vaccination. Additionally, the conversation regarding the infrastructure and preparedness is not complete without acknowledging the vital role that community health workers have in healthcare delivery in African nations. The valuable connections that community health workers have in their communities, coupled with their skills and expertise, could be leveraged to strengthen the public health response to COVID-19 in African nations and reduce vaccine hesitancy.^13^ These existing systems could be used to educate local populations about COVID-19 and disseminate vaccines.

Africa is an heterogenous continent. Overall, the pandemic has strained already vulnerable healthcare systems, widened economic inequalities, and exacerbated health disparities.^14^ As of April 30, 2021, more than 4.5 million cases of COVID-19 and 120,000 deaths have been reported in Africa.^15^ However, the impact of the pandemic has varied greatly among nations.^16^^,^^17^ The current COVAX plan, as originally conceived, failed to account for these circumstances and does not incorporate the World Health Organization’s Strategic Advisory Group of Experts on Immunization (WHO SAGE) value framework into its model. The SAGE value framework, which was formed to provide guidance regarding the allocation of COVID-19 vaccines both globally and nationally, recommends that equitable allocation and prioritization of vaccines should take into consideration the special epidemic risks and needs of participating nations.^18^ Utilization of this framework is especially important in Africa, where there are clinical deserts, weak public health infrastructures, and limited social resources. Lessons learned from previous Ebola epidemics have demonstrated how a public health crisis in politically and economically fragile nations can disrupt systems beyond healthcare and how carefully targeted vaccine campaigns can be mounted. The 2013 to 2016 Ebola outbreak in Liberia, Sierra Leone, and Guinea resulted in the deaths of 513 healthcare workers in these countries,^19^ equating to losses of 8%, 7%, and 1% of the healthcare workforce, respectively,^20^ as well as $2.8 billion in lost revenue from disruptions in mining, agriculture, tourism, trade, and transportation sectors.^21^ The aftermath of the Ebola outbreak demonstrated why it is pivotal for COVAX to take a tailored approach to vaccine distribution by factoring in the infrastructure needs unique to each nation.

It is justifiable for COVAX and the global community to vaccinate 100% of Africa. History tells us this vision is feasible now, and it also reminds us that there will be future pandemics. After the Ebola epidemic ended in 2016, the world’s financial commitments to build a stronger health system and prevent the next emerging pandemic were forgotten. Before COVAX dissolves, it should be leveraged to create the most long-term and sustainable healthcare developments possible for Africa. If this time-sensitive opportunity is missed, Africa will remain the persisting frontier of the underdeveloped health system where viruses will mutate into more dangerous forms. Emerging pandemics of newly discovered viruses are costly, deadly, and difficult to control. We see two options: pay now or pay later. We cannot put a price tag on a life, so we consider the former option, pay now (before unnecessary lives are lost), as the principled approach.
